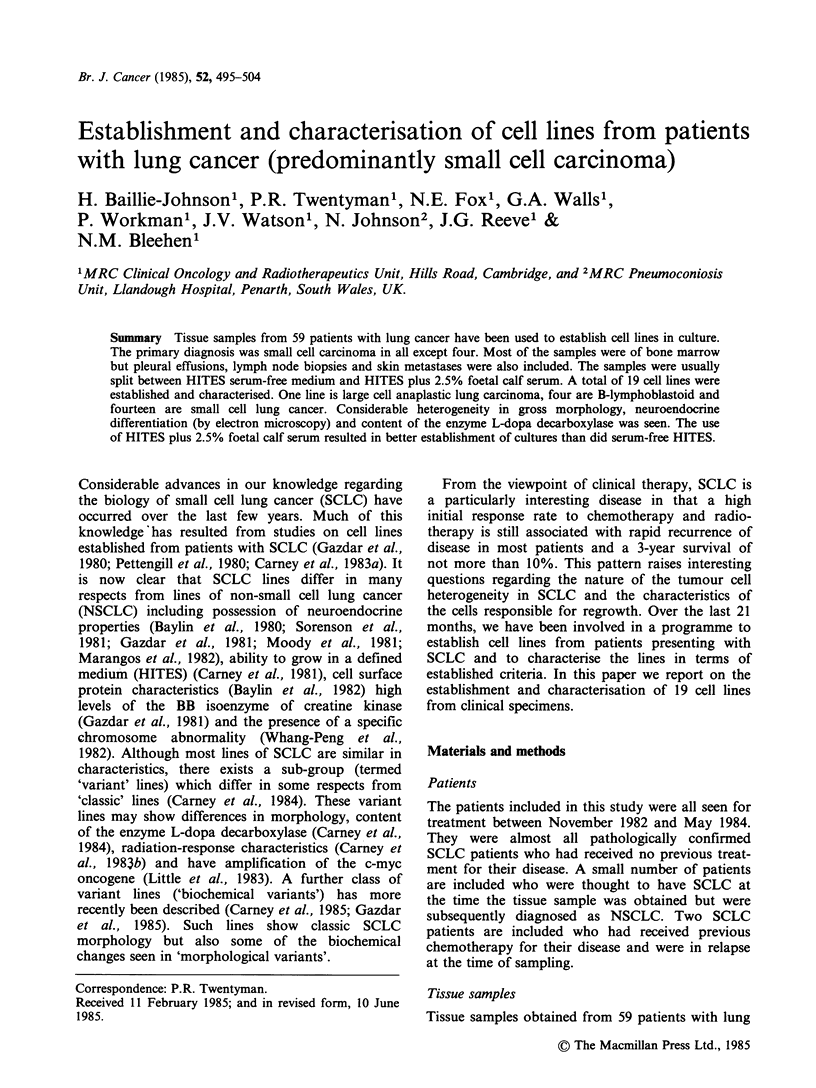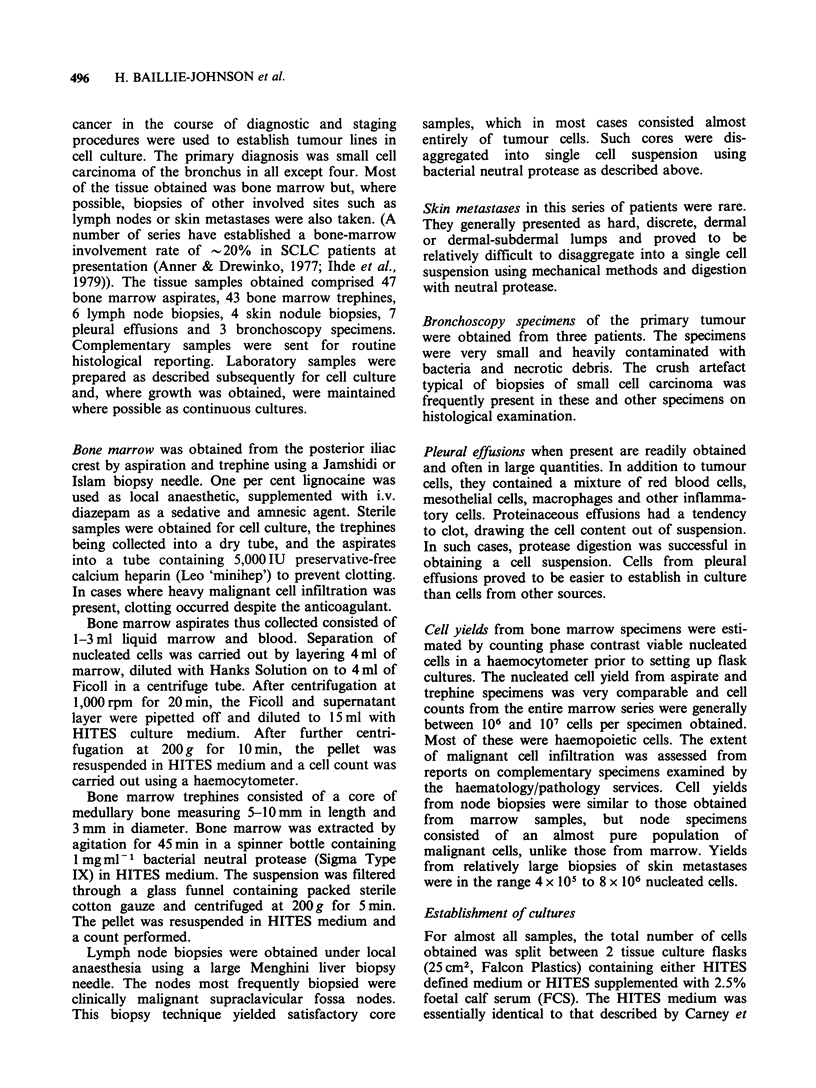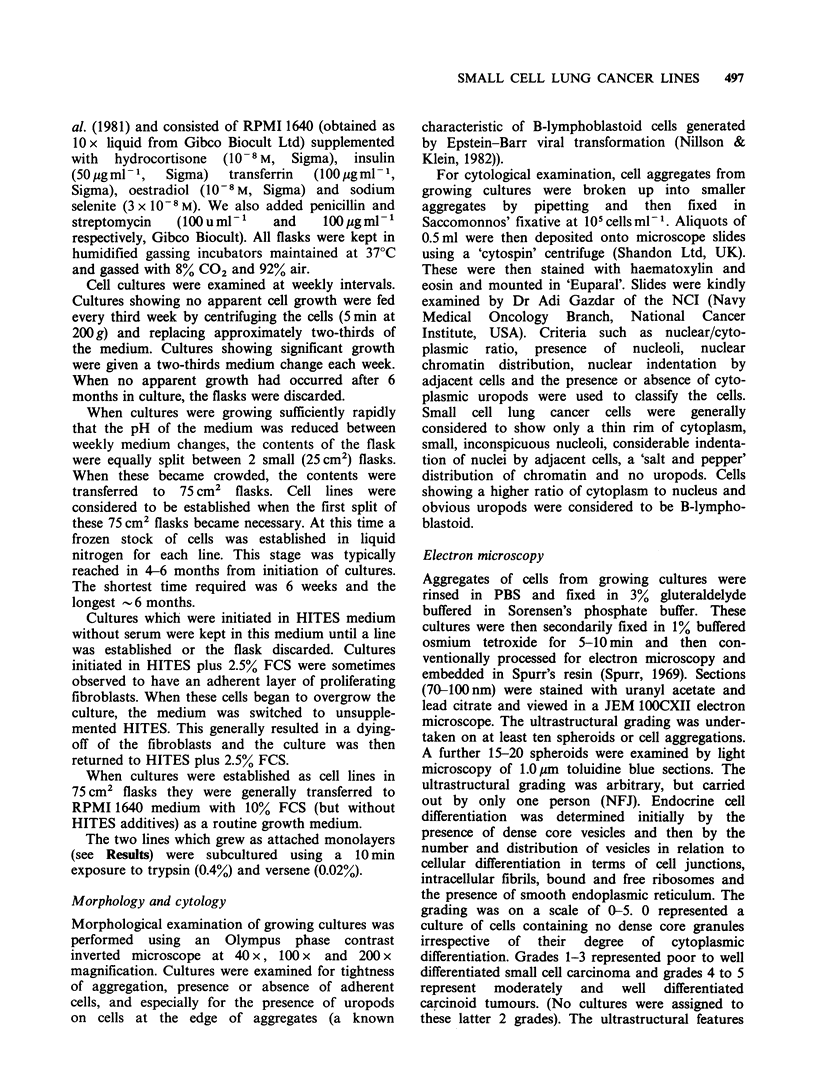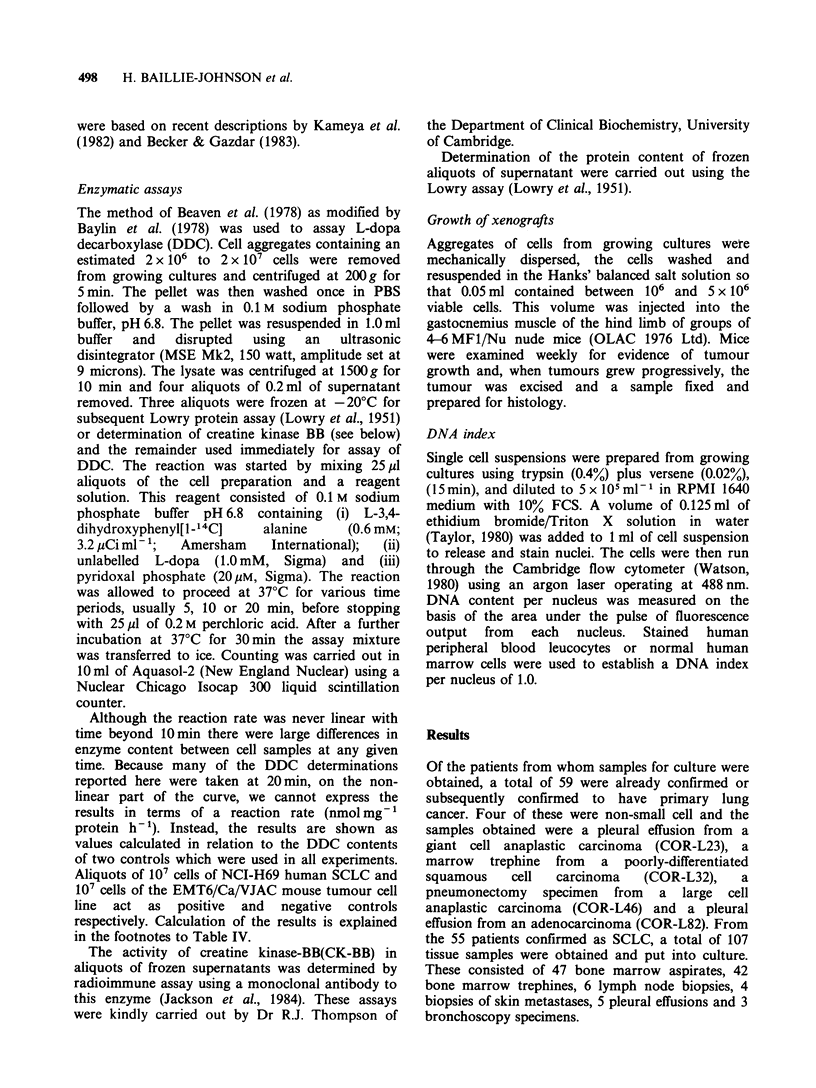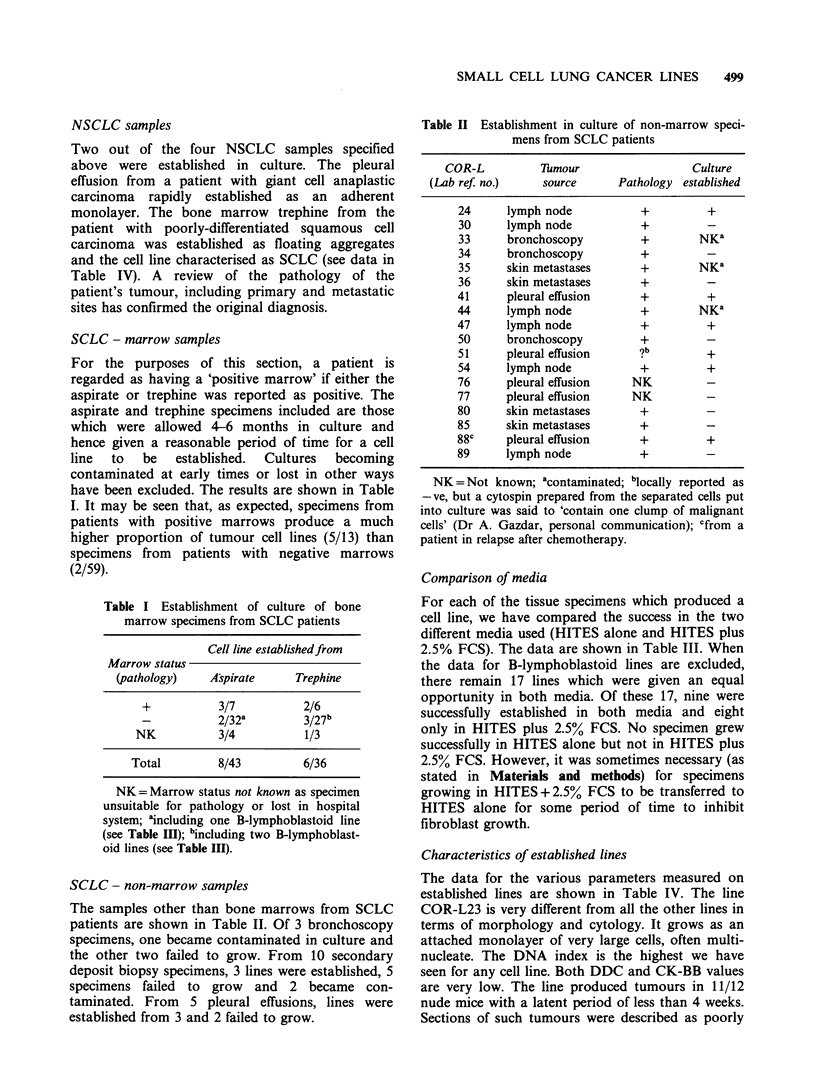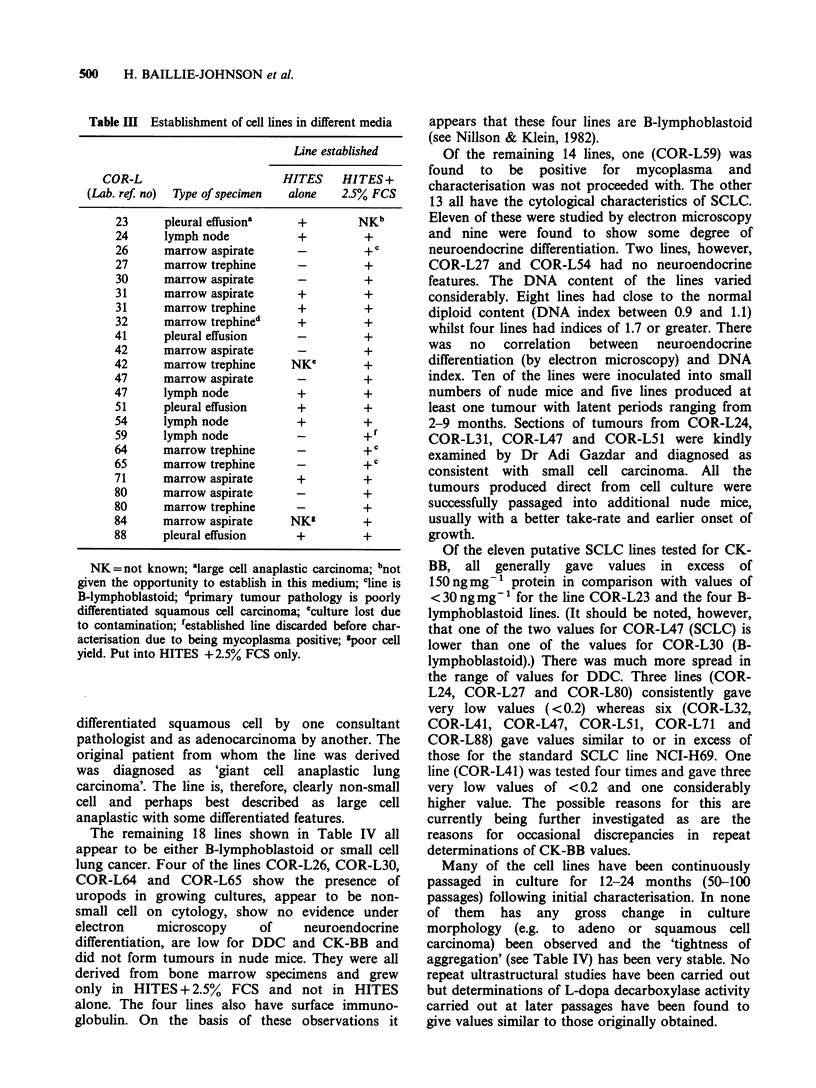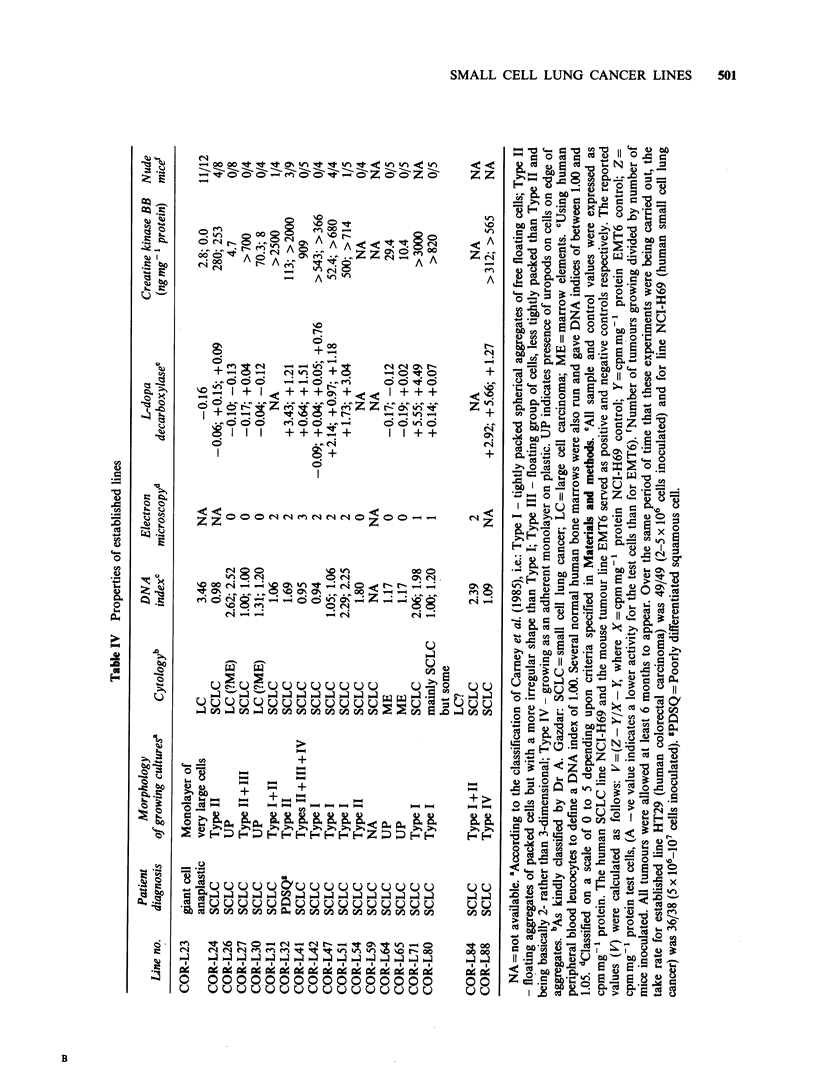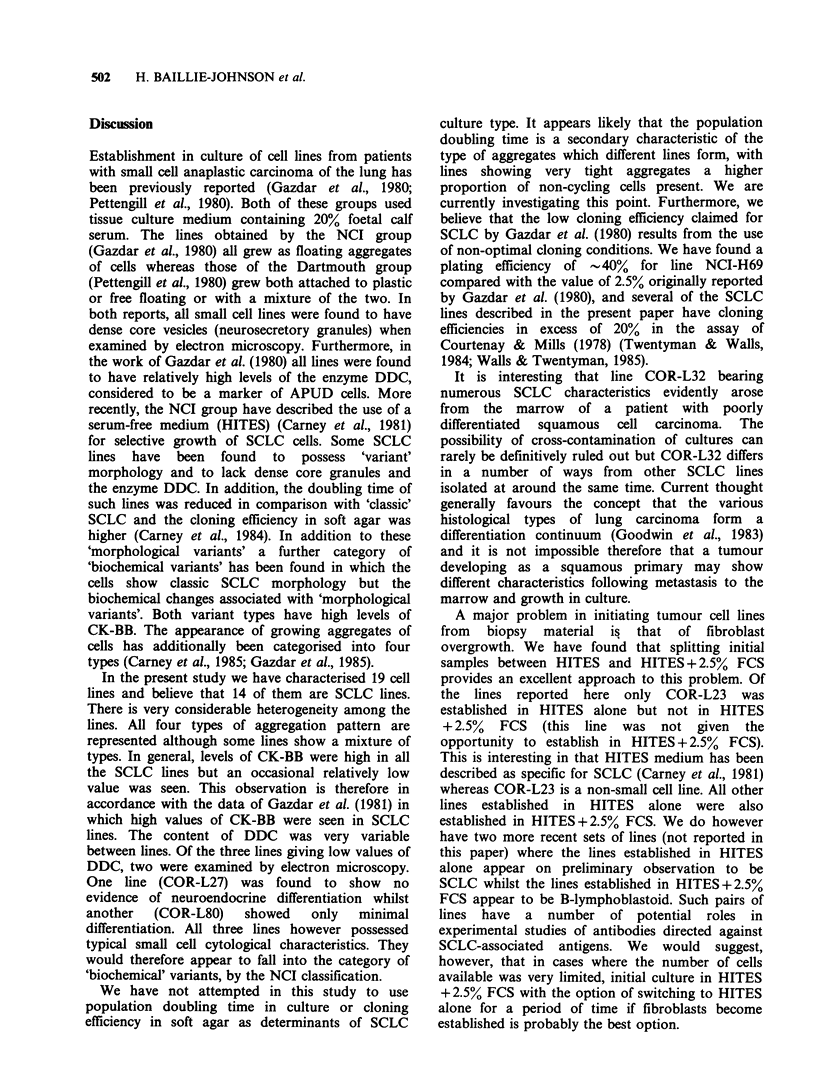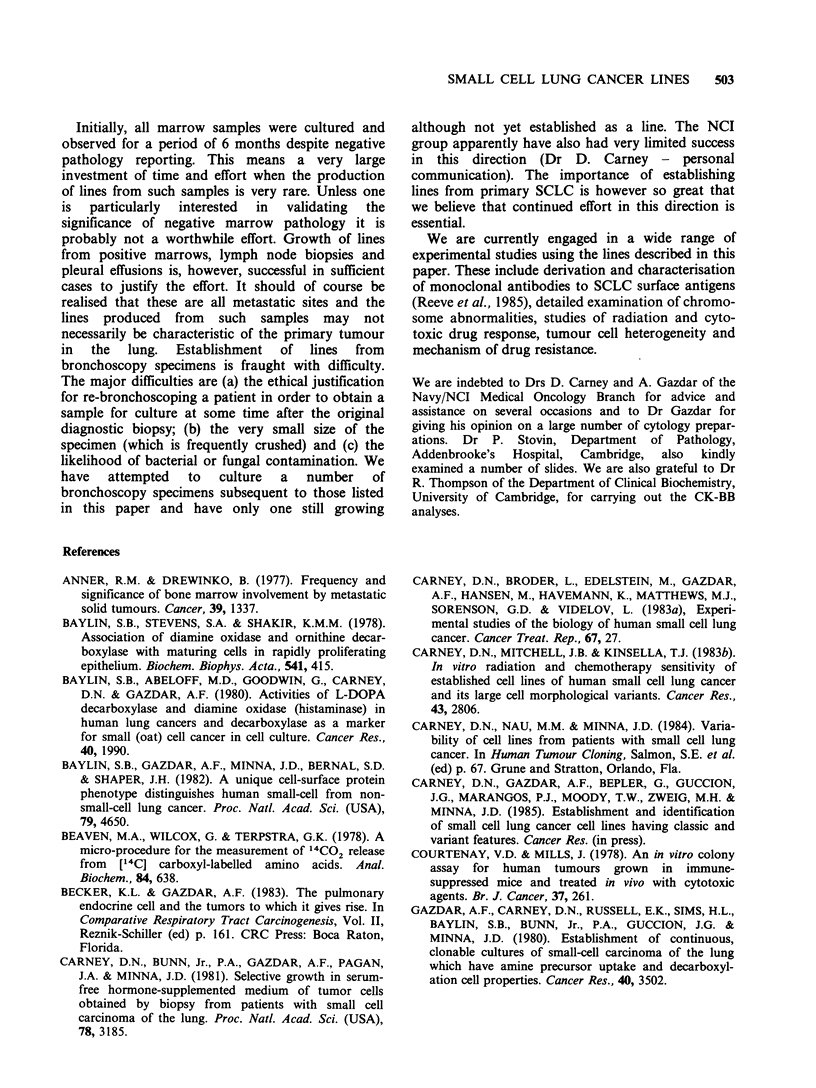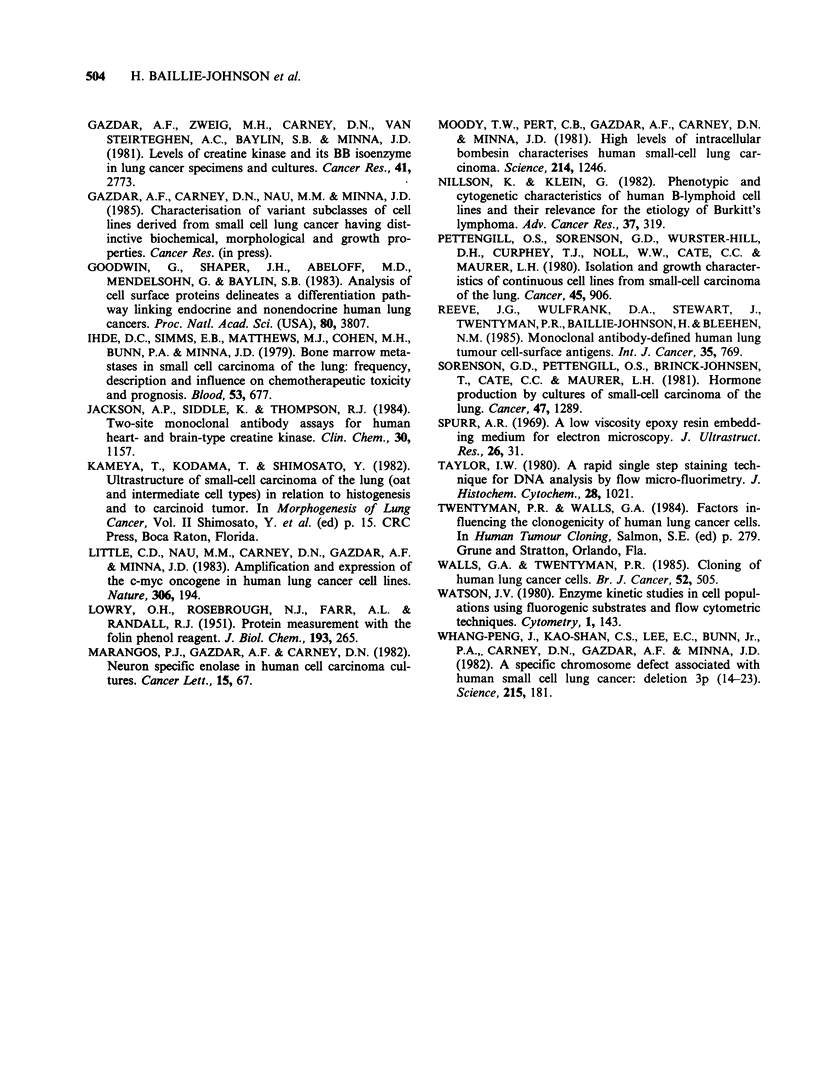# Establishment and characterisation of cell lines from patients with lung cancer (predominantly small cell carcinoma).

**DOI:** 10.1038/bjc.1985.220

**Published:** 1985-10

**Authors:** H. Baillie-Johnson, P. R. Twentyman, N. E. Fox, G. A. Walls, P. Workman, J. V. Watson, N. Johnson, J. G. Reeve, N. M. Bleehen

## Abstract

Tissue samples from 59 patients with lung cancer have been used to establish cell lines in culture. The primary diagnosis was small cell carcinoma in all except four. Most of the samples were of bone marrow but pleural effusions, lymph node biopsies and skin metastases were also included. The samples were usually split between HITES serum-free medium and HITES plus 2.5% foetal calf serum. A total of 19 cell lines were established and characterised. One line is large cell anaplastic lung carcinoma, four are B-lymphoblastoid and fourteen are small cell lung cancer. Considerable heterogeneity in gross morphology, neuroendocrine differentiation (by electron microscopy) and content of the enzyme L-dopa decarboxylase was seen. The use of HITES plus 2.5% foetal calf serum resulted in better establishment of cultures than did serum-free HITES.


Br. J. Cancer (1985), 52, 495–504

### Establishment and characterisation of cell lines from patients with lung cancer (predominantly small cell carcinoma)

H. Baillie-Johnson[1], P.R. Twentyman[1], N.E. Fox[1], G.A. Walls[1], P. Workman[1], J.V. Watson[1], N. Johnson[2], J.G. Reeve[1] & N.M. Bleehen[1]

[1]*MRC Clinical Oncology and Radiotherapeutics Unit, Hills Road, Cambridge, and* [2]*MRC Pneumoconiosis Unit, Llandough Hospital, Penarth, South Wales, UK.*

**Summary** Tissue samples from 59 patients with lung cancer have been used to establish cell lines in culture. The primary diagnosis was small cell carcinoma in all except four. Most of the samples were of bone marrow but pleural effusions, lymph node biopsies and skin metastases were also included. The samples were usually split between HITES serum-free medium and HITES plus 2.5% foetal calf serum. A total of 19 cell lines were established and characterised. One line is large cell anaplastic lung carcinoma, four are B-lymphoblastoid and fourteen are small cell lung cancer. Considerable heterogeneity in gross morphology, neuroendocrine differentiation (by electron microscopy) and content of the enzyme L-dopa decarboxylase was seen. The use of HITES plus 2.5% foetal calf serum resulted in better establishment of cultures than did serum-free HITES.

Considerable advances in our knowledge regarding the biology of small cell lung cancer (SCLC) have occurred over the last few years. Much of this knowledge has resulted from studies on cell lines established from patients with SCLC (Gazdar *et al.*, 1980; Pettengill *et al.*, 1980; Carney *et al.*, 1983*a*). It is now clear that SCLC lines differ in many respects from lines of non-small cell lung cancer (NSCLC) including possession of neuroendocrine properties (Baylin *et al.*, 1980; Sorenson *et al.*, 1981; Gazdar *et al.*, 1981; Moody *et al.*, 1981; Marangos *et al.*, 1982), ability to grow in a defined medium (HITES) (Carney *et al.*, 1981), cell surface protein characteristics (Baylin *et al.*, 1982) high levels of the BB isoenzyme of creatine kinase (Gazdar *et al.*, 1981) and the presence of a specific chromosome abnormality (Whang-Peng *et al.*, 1982). Although most lines of SCLC are similar in characteristics, there exists a sub-group (termed 'variant' lines) which differ in some respects from 'classic' lines (Carney *et al.*, 1984). These variant lines may show differences in morphology, content of the enzyme L-dopa decarboxylase (Carney *et al.*, 1984), radiation-response characteristics (Carney *et al.*, 1983*b*) and have amplification of the c-myc oncogene (Little *et al.*, 1983). A further class of variant lines ('biochemical variants') has more recently been described (Carney *et al.*, 1985; Gazdar *et al.*, 1985). Such lines show classic SCLC morphology but also some of the biochemical changes seen in 'morphological variants'.

Correspondence: P.R. Twentyman.
Received 11 February 1985; and in revised form, 10 June 1985.

From the viewpoint of clinical therapy, SCLC is a particularly interesting disease in that a high initial response rate to chemotherapy and radiotherapy is still associated with rapid recurrence of disease in most patients and a 3-year survival of not more than 10%. This pattern raises interesting questions regarding the nature of the tumour cell heterogeneity in SCLC and the characteristics of the cells responsible for regrowth. Over the last 21 months, we have been involved in a programme to establish cell lines from patients presenting with SCLC and to characterise the lines in terms of established criteria. In this paper we report on the establishment and characterisation of 19 cell lines from clinical specimens.

### Materials and methods

### *Patients*

The patients included in this study were all seen for treatment between November 1982 and May 1984. They were almost all pathologically confirmed SCLC patients who had received no previous treatment for their disease. A small number of patients are included who were thought to have SCLC at the time the tissue sample was obtained but were subsequently diagnosed as NSCLC. Two SCLC patients are included who had received previous chemotherapy for their disease and were in relapse at the time of sampling.

### *Tissue samples*

Tissue samples obtained from 59 patients with lung

© The Macmillan Press Ltd., 1985

cancer in the course of diagnostic and staging procedures were used to establish tumour lines in cell culture. The primary diagnosis was small cell carcinoma of the bronchus in all except four. Most of the tissue obtained was bone marrow but, where possible, biopsies of other involved sites such as lymph nodes or skin metastases were also taken. (A number of series have established a bone-marrow involvement rate of ~20% in SCLC patients at presentation (Anner & Drewinko, 1977; Ihde et al., 1979)). The tissue samples obtained comprised 47 bone marrow aspirates, 43 bone marrow trephines, 6 lymph node biopsies, 4 skin nodule biopsies, 7 pleural effusions and 3 bronchoscopy specimens. Complementary samples were sent for routine histological reporting. Laboratory samples were prepared as described subsequently for cell culture and, where growth was obtained, were maintained where possible as continuous cultures.

*Bone marrow* was obtained from the posterior iliac crest by aspiration and trephine using a Jamshidi or Islam biopsy needle. One per cent lignocaine was used as local anaesthetic, supplemented with i.v. diazepam as a sedative and amnesic agent. Sterile samples were obtained for cell culture, the trephines being collected into a dry tube, and the aspirates into a tube containing 5,000 IU preservative-free calcium heparin (Leo 'minihep') to prevent clotting. In cases where heavy malignant cell infiltration was present, clotting occurred despite the anticoagulant.

Bone marrow aspirates thus collected consisted of 1–3 ml liquid marrow and blood. Separation of nucleated cells was carried out by layering 4 ml of marrow, diluted with Hanks Solution on to 4 ml of Ficoll in a centrifuge tube. After centrifugation at 1,000 rpm for 20 min, the Ficoll and supernatant layer were pipetted off and diluted to 15 ml with HITES culture medium. After further centrifugation at 200 *g* for 10 min, the pellet was resuspended in HITES medium and a cell count was carried out using a haemocytometer.

Bone marrow trephines consisted of a core of medullary bone measuring 5–10 mm in length and 3 mm in diameter. Bone marrow was extracted by agitation for 45 min in a spinner bottle containing 1 mg $ml^{-1}$ bacterial neutral protease (Sigma Type IX) in HITES medium. The suspension was filtered through a glass funnel containing packed sterile cotton gauze and centrifuged at 200 *g* for 5 min. The pellet was resuspended in HITES medium and a count performed.

Lymph node biopsies were obtained under local anaesthesia using a large Menghini liver biopsy needle. The nodes most frequently biopsied were clinically malignant supraclavicular fossa nodes. This biopsy technique yielded satisfactory core samples, which in most cases consisted almost entirely of tumour cells. Such cores were disaggregated into single cell suspension using bacterial neutral protease as described above.

*Skin metastases* in this series of patients were rare. They generally presented as hard, discrete, dermal or dermal-subdermal lumps and proved to be relatively difficult to disaggregate into a single cell suspension using mechanical methods and digestion with neutral protease.

*Bronchoscopy specimens* of the primary tumour were obtained from three patients. The specimens were very small and heavily contaminated with bacteria and necrotic debris. The crush artefact typical of biopsies of small cell carcinoma was frequently present in these and other specimens on histological examination.

*Pleural effusions* when present are readily obtained and often in large quantities. In addition to tumour cells, they contained a mixture of red blood cells, mesothelial cells, macrophages and other inflammatory cells. Proteinaceous effusions had a tendency to clot, drawing the cell content out of suspension. In such cases, protease digestion was successful in obtaining a cell suspension. Cells from pleural effusions proved to be easier to establish in culture than cells from other sources.

*Cell yields* from bone marrow specimens were estimated by counting phase contrast viable nucleated cells in a haemocytometer prior to setting up flask cultures. The nucleated cell yield from aspirate and trephine specimens was very comparable and cell counts from the entire marrow series were generally between $10^6$ and $10^7$ cells per specimen obtained. Most of these were haemopoietic cells. The extent of malignant cell infiltration was assessed from reports on complementary specimens examined by the haematology/pathology services. Cell yields from node biopsies were similar to those obtained from marrow samples, but node specimens consisted of an almost pure population of malignant cells, unlike those from marrow. Yields from relatively large biopsies of skin metastases were in the range $4 \times 10^5$ to $8 \times 10^6$ nucleated cells.

### *Establishment of cultures*

For almost all samples, the total number of cells obtained was split between 2 tissue culture flasks (25 $cm^2$, Falcon Plastics) containing either HITES defined medium or HITES supplemented with 2.5% foetal calf serum (FCS). The HITES medium was essentially identical to that described by Carney *et*

*al.* (1981) and consisted of RPMI 1640 (obtained as 10× liquid from Gibco Biocult Ltd) supplemented with hydrocortisone ($10^{-8}$ M, Sigma), insulin (50 $\mu$g ml$^{-1}$, Sigma) transferrin (100 $\mu$g ml$^{-1}$, Sigma), oestradiol ($10^{-8}$ M, Sigma) and sodium selenite ($3 \times 10^{-8}$ M). We also added penicillin and streptomycin (100 u ml$^{-1}$ and 100 $\mu$g ml$^{-1}$ respectively, Gibco Biocult). All flasks were kept in humidified gassing incubators maintained at 37°C and gassed with 8% $CO_2$ and 92% air.

Cell cultures were examined at weekly intervals. Cultures showing no apparent cell growth were fed every third week by centrifuging the cells (5 min at 200 *g*) and replacing approximately two-thirds of the medium. Cultures showing significant growth were given a two-thirds medium change each week. When no apparent growth had occurred after 6 months in culture, the flasks were discarded.

When cultures were growing sufficiently rapidly that the pH of the medium was reduced between weekly medium changes, the contents of the flask were equally split between 2 small (25 cm$^2$) flasks. When these became crowded, the contents were transferred to 75 cm$^2$ flasks. Cell lines were considered to be established when the first split of these 75 cm$^2$ flasks became necessary. At this time a frozen stock of cells was established in liquid nitrogen for each line. This stage was typically reached in 4–6 months from initiation of cultures. The shortest time required was 6 weeks and the longest ~6 months.

Cultures which were initiated in HITES medium without serum were kept in this medium until a line was established or the flask discarded. Cultures initiated in HITES plus 2.5% FCS were sometimes observed to have an adherent layer of proliferating fibroblasts. When these cells began to overgrow the culture, the medium was switched to unsupplemented HITES. This generally resulted in a dying-off of the fibroblasts and the culture was then returned to HITES plus 2.5% FCS.

When cultures were established as cell lines in 75 cm$^2$ flasks they were generally transferred to RPMI 1640 medium with 10% FCS (but without HITES additives) as a routine growth medium.

The two lines which grew as attached monolayers (see **Results**) were subcultured using a 10 min exposure to trypsin (0.4%) and versene (0.02%).

*Morphology and cytology*

Morphological examination of growing cultures was performed using an Olympus phase contrast inverted microscope at 40×, 100× and 200× magnification. Cultures were examined for tightness of aggregation, presence or absence of adherent cells, and especially for the presence of uropods on cells at the edge of aggregates (a known characteristic of B-lymphoblastoid cells generated by Epstein–Barr viral transformation (Nillson & Klein, 1982)).

For cytological examination, cell aggregates from growing cultures were broken up into smaller aggregates by pipetting and then fixed in Saccomonnos' fixative at $10^5$ cells ml$^{-1}$. Aliquots of 0.5 ml were then deposited onto microscope slides using a 'cytospin' centrifuge (Shandon Ltd, UK). These were then stained with haematoxylin and eosin and mounted in 'Euparal'. Slides were kindly examined by Dr Adi Gazdar of the NCI (Navy Medical Oncology Branch, National Cancer Institute, USA). Criteria such as nuclear/cytoplasmic ratio, presence of nucleoli, nuclear chromatin distribution, nuclear indentation by adjacent cells and the presence or absence of cytoplasmic uropods were used to classify the cells. Small cell lung cancer cells were generally considered to show only a thin rim of cytoplasm, small, inconspicuous nucleoli, considerable indentation of nuclei by adjacent cells, a 'salt and pepper' distribution of chromatin and no uropods. Cells showing a higher ratio of cytoplasm to nucleus and obvious uropods were considered to be B-lymphoblastoid.

*Electron microscopy*

Aggregates of cells from growing cultures were rinsed in PBS and fixed in 3% gluteraldelyde buffered in Sorensen's phosphate buffer. These cultures were then secondarily fixed in 1% buffered osmium tetroxide for 5–10 min and then conventionally processed for electron microscopy and embedded in Spurr's resin (Spurr, 1969). Sections (70–100 nm) were stained with uranyl acetate and lead citrate and viewed in a JEM 100CXII electron microscope. The ultrastructural grading was undertaken on at least ten spheroids or cell aggregations. A further 15–20 spheroids were examined by light microscopy of 1.0 $\mu$m toluidine blue sections. The ultrastructural grading was arbitrary, but carried out by only one person (NFJ). Endocrine cell differentiation was determined initially by the presence of dense core vesicles and then by the number and distribution of vesicles in relation to cellular differentiation in terms of cell junctions, intracellular fibrils, bound and free ribosomes and the presence of smooth endoplasmic reticulum. The grading was on a scale of 0–5. 0 represented a culture of cells containing no dense core granules irrespective of their degree of cytoplasmic differentiation. Grades 1–3 represented poor to well differentiated small cell carcinoma and grades 4 to 5 represent moderately and well differentiated carcinoid tumours. (No cultures were assigned to these latter 2 grades). The ultrastructural features were based on recent descriptions by Kameya et al. (1982) and Becker & Gazdar (1983).

*Enzymatic assays*

The method of Beaven et al. (1978) as modified by Baylin et al. (1978) was used to assay L-dopa decarboxylase (DDC). Cell aggregates containing an estimated $2 \times 10^6$ to $2 \times 10^7$ cells were removed from growing cultures and centrifuged at 200 g for 5 min. The pellet was then washed once in PBS followed by a wash in 0.1 M sodium phosphate buffer, pH 6.8. The pellet was resuspended in 1.0 ml buffer and disrupted using an ultrasonic disintegrator (MSE Mk2, 150 watt, amplitude set at 9 microns). The lysate was centrifuged at 1500 g for 10 min and four aliquots of 0.2 ml of supernatant removed. Three aliquots were frozen at −20°C for subsequent Lowry protein assay (Lowry et al., 1951) or determination of creatine kinase BB (see below) and the remainder used immediately for assay of DDC. The reaction was started by mixing 25 $\mu$l aliquots of the cell preparation and a reagent solution. This reagent consisted of 0.1 M sodium phosphate buffer pH 6.8 containing (i) L-3,4-dihydroxyphenyl[1-$^{14}$C] alanine (0.6 mM; 3.2 $\mu$Ci ml$^{-1}$; Amersham International); (ii) unlabelled L-dopa (1.0 mM, Sigma) and (iii) pyridoxal phosphate (20 $\mu$M, Sigma). The reaction was allowed to proceed at 37°C for various time periods, usually 5, 10 or 20 min, before stopping with 25 $\mu$l of 0.2 M perchloric acid. After a further incubation at 37°C for 30 min the assay mixture was transferred to ice. Counting was carried out in 10 ml of Aquasol-2 (New England Nuclear) using a Nuclear Chicago Isocap 300 liquid scintillation counter.

Although the reaction rate was never linear with time beyond 10 min there were large differences in enzyme content between cell samples at any given time. Because many of the DDC determinations reported here were taken at 20 min, on the non-linear part of the curve, we cannot express the results in terms of a reaction rate (nmol mg$^{-1}$ protein h$^{-1}$). Instead, the results are shown as values calculated in relation to the DDC contents of two controls which were used in all experiments. Aliquots of $10^7$ cells of NCI-H69 human SCLC and $10^7$ cells of the EMT6/Ca/VJAC mouse tumour cell line act as positive and negative controls respectively. Calculation of the results is explained in the footnotes to Table IV.

The activity of creatine kinase-BB(CK-BB) in aliquots of frozen supernatants was determined by radioimmune assay using a monoclonal antibody to this enzyme (Jackson et al., 1984). These assays were kindly carried out by Dr R.J. Thompson of the Department of Clinical Biochemistry, University of Cambridge.

Determination of the protein content of frozen aliquots of supernatant were carried out using the Lowry assay (Lowry et al., 1951).

*Growth of xenografts*

Aggregates of cells from growing cultures were mechanically dispersed, the cells washed and resuspended in the Hanks' balanced salt solution so that 0.05 ml contained between $10^6$ and $5 \times 10^6$ viable cells. This volume was injected into the gastocnemius muscle of the hind limb of groups of 4–6 MF1/Nu nude mice (OLAC 1976 Ltd). Mice were examined weekly for evidence of tumour growth and, when tumours grew progressively, the tumour was excised and a sample fixed and prepared for histology.

*DNA index*

Single cell suspensions were prepared from growing cultures using trypsin (0.4%) plus versene (0.02%), (15 min), and diluted to $5 \times 10^5$ ml$^{-1}$ in RPMI 1640 medium with 10% FCS. A volume of 0.125 ml of ethidium bromide/Triton X solution in water (Taylor, 1980) was added to 1 ml of cell suspension to release and stain nuclei. The cells were then run through the Cambridge flow cytometer (Watson, 1980) using an argon laser operating at 488 nm. DNA content per nucleus was measured on the basis of the area under the pulse of fluorescence output from each nucleus. Stained human peripheral blood leucocytes or normal human marrow cells were used to establish a DNA index per nucleus of 1.0.

### Results

Of the patients from whom samples for culture were obtained, a total of 59 were already confirmed or subsequently confirmed to have primary lung cancer. Four of these were non-small cell and the samples obtained were a pleural effusion from a giant cell anaplastic carcinoma (COR-L23), a marrow trephine from a poorly-differentiated squamous cell carcinoma (COR-L32), a pneumonectomy specimen from a large cell anaplastic carcinoma (COR-L46) and a pleural effusion from an adenocarcinoma (COR-L82). From the 55 patients confirmed as SCLC, a total of 107 tissue samples were obtained and put into culture. These consisted of 47 bone marrow aspirates, 42 bone marrow trephines, 6 lymph node biopsies, 4 biopsies of skin metastases, 5 pleural effusions and 3 bronchoscopy specimens.

*NSCLC samples*

Two out of the four NSCLC samples specified above were established in culture. The pleural effusion from a patient with giant cell anaplastic carcinoma rapidly established as an adherent monolayer. The bone marrow trephine from the patient with poorly-differentiated squamous cell carcinoma was established as floating aggregates and the cell line characterised as SCLC (see data in Table IV). A review of the pathology of the patient's tumour, including primary and metastatic sites has confirmed the original diagnosis.

*SCLC – marrow samples*

For the purposes of this section, a patient is regarded as having a 'positive marrow' if either the aspirate or trephine was reported as positive. The aspirate and trephine specimens included are those which were allowed 4–6 months in culture and hence given a reasonable period of time for a cell line to be established. Cultures becoming contaminated at early times or lost in other ways have been excluded. The results are shown in Table I. It may be seen that, as expected, specimens from patients with positive marrows produce a much higher proportion of tumour cell lines (5/13) than specimens from patients with negative marrows (2/59).

**Table I** Establishment of culture of bone marrow specimens from SCLC patients

| Marrow status (pathology) | Cell line established from | |
|---|---|---|
| | *Aspirate* | *Trephine* |
| + | 3/7 | 2/6 |
| – | 2/32[a] | 3/27[b] |
| NK | 3/4 | 1/3 |
| Total | 8/43 | 6/36 |

NK = Marrow status *not known* as specimen unsuitable for pathology or lost in hospital system; [a]including one B-lymphoblastoid line (see **Table III**); [b]including two B-lymphoblastoid lines (see **Table III**).

*SCLC – non-marrow samples*

The samples other than bone marrows from SCLC patients are shown in Table II. Of 3 bronchoscopy specimens, one became contaminated in culture and the other two failed to grow. From 10 secondary deposit biopsy specimens, 3 lines were established, 5 specimens failed to grow and 2 became contaminated. From 5 pleural effusions, lines were established from 3 and 2 failed to grow.

**Table II** Establishment in culture of non-marrow specimens from SCLC patients

| *COR-L (Lab ref. no.)* | *Tumour source* | *Pathology* | *Culture established* |
|---|---|---|---|
| 24 | lymph node | + | + |
| 30 | lymph node | + | – |
| 33 | bronchoscopy | + | NK[a] |
| 34 | bronchoscopy | + | – |
| 35 | skin metastases | + | NK[a] |
| 36 | skin metastases | + | – |
| 41 | pleural effusion | + | + |
| 44 | lymph node | + | NK[a] |
| 47 | lymph node | + | + |
| 50 | bronchoscopy | + | – |
| 51 | pleural effusion | ?[b] | + |
| 54 | lymph node | + | + |
| 76 | pleural effusion | NK | – |
| 77 | pleural effusion | NK | – |
| 80 | skin metastases | + | – |
| 85 | skin metastases | + | – |
| 88[c] | pleural effusion | + | + |
| 89 | lymph node | + | – |

NK = Not known; [a]contaminated; [b]locally reported as –ve, but a cytospin prepared from the separated cells put into culture was said to 'contain one clump of malignant cells' (Dr A. Gazdar, personal communication); [c]from a patient in relapse after chemotherapy.

*Comparison of media*

For each of the tissue specimens which produced a cell line, we have compared the success in the two different media used (HITES alone and HITES plus 2.5% FCS). The data are shown in Table III. When the data for B-lymphoblastoid lines are excluded, there remain 17 lines which were given an equal opportunity in both media. Of these 17, nine were successfully established in both media and eight only in HITES plus 2.5% FCS. No specimen grew successfully in HITES alone but not in HITES plus 2.5% FCS. However, it was sometimes necessary (as stated in **Materials and methods**) for specimens growing in HITES + 2.5% FCS to be transferred to HITES alone for some period of time to inhibit fibroblast growth.

*Characteristics of established lines*

The data for the various parameters measured on established lines are shown in Table IV. The line COR-L23 is very different from all the other lines in terms of morphology and cytology. It grows as an attached monolayer of very large cells, often multinucleate. The DNA index is the highest we have seen for any cell line. Both DDC and CK-BB values are very low. The line produced tumours in 11/12 nude mice with a latent period of less than 4 weeks. Sections of such tumours were described as poorly

**Table III** Establishment of cell lines in different media

| COR-L (Lab. ref. no) | Type of specimen | Line established: HITES alone | Line established: HITES+ 2.5% FCS |
|---|---|---|---|
| 23 | pleural effusion[a] | + | NK[b] |
| 24 | lymph node | + | + |
| 26 | marrow aspirate | − | +[c] |
| 27 | marrow trephine | − | + |
| 30 | marrow aspirate | − | + |
| 31 | marrow aspirate | + | + |
| 31 | marrow trephine | + | + |
| 32 | marrow trephine[d] | + | + |
| 41 | pleural effusion | − | + |
| 42 | marrow aspirate | − | + |
| 42 | marrow trephine | NK[e] | + |
| 47 | marrow aspirate | − | + |
| 47 | lymph node | + | + |
| 51 | pleural effusion | + | + |
| 54 | lymph node | + | + |
| 59 | lymph node | − | +[f] |
| 64 | marrow trephine | − | +[c] |
| 65 | marrow trephine | − | +[c] |
| 71 | marrow aspirate | + | + |
| 80 | marrow aspirate | − | + |
| 80 | marrow trephine | − | + |
| 84 | marrow aspirate | NK[g] | + |
| 88 | pleural effusion | + | + |

NK = not known; [a]large cell anaplastic carcinoma; [b]not given the opportunity to establish in this medium; [c]line is B-lymphoblastoid; [d]primary tumour pathology is poorly differentiated squamous cell carcinoma; [e]culture lost due to contamination; [f]established line discarded before characterisation due to being mycoplasma positive; [g]poor cell yield. Put into HITES +2.5% FCS only.

differentiated squamous cell by one consultant pathologist and as adenocarcinoma by another. The original patient from whom the line was derived was diagnosed as 'giant cell anaplastic lung carcinoma'. The line is, therefore, clearly non-small cell and perhaps best described as large cell anaplastic with some differentiated features.

The remaining 18 lines shown in Table IV all appear to be either B-lymphoblastoid or small cell lung cancer. Four of the lines COR-L26, COR-L30, COR-L64 and COR-L65 show the presence of uropods in growing cultures, appear to be non-small cell on cytology, show no evidence under electron microscopy of neuroendocrine differentiation, are low for DDC and CK-BB and did not form tumours in nude mice. They were all derived from bone marrow specimens and grew only in HITES+2.5% FCS and not in HITES alone. The four lines also have surface immunoglobulin. On the basis of these observations it appears that these four lines are B-lymphoblastoid (see Nillson & Klein, 1982).

Of the remaining 14 lines, one (COR-L59) was found to be positive for mycoplasma and characterisation was not proceeded with. The other 13 all have the cytological characteristics of SCLC. Eleven of these were studied by electron microscopy and nine were found to show some degree of neuroendocrine differentiation. Two lines, however, COR-L27 and COR-L54 had no neuroendocrine features. The DNA content of the lines varied considerably. Eight lines had close to the normal diploid content (DNA index between 0.9 and 1.1) whilst four lines had indices of 1.7 or greater. There was no correlation between neuroendocrine differentiation (by electron microscopy) and DNA index. Ten of the lines were inoculated into small numbers of nude mice and five lines produced at least one tumour with latent periods ranging from 2–9 months. Sections of tumours from COR-L24, COR-L31, COR-L47 and COR-L51 were kindly examined by Dr Adi Gazdar and diagnosed as consistent with small cell carcinoma. All the tumours produced direct from cell culture were successfully passaged into additional nude mice, usually with a better take-rate and earlier onset of growth.

Of the eleven putative SCLC lines tested for CK-BB, all generally gave values in excess of 150 ng mg$^{-1}$ protein in comparison with values of <30 ng mg$^{-1}$ for the line COR-L23 and the four B-lymphoblastoid lines. (It should be noted, however, that one of the two values for COR-L47 (SCLC) is lower than one of the values for COR-L30 (B-lymphoblastoid).) There was much more spread in the range of values for DDC. Three lines (COR-L24, COR-L27 and COR-L80) consistently gave very low values (<0.2) whereas six (COR-L32, COR-L41, COR-L47, COR-L51, COR-L71 and COR-L88) gave values similar to or in excess of those for the standard SCLC line NCI-H69. One line (COR-L41) was tested four times and gave three very low values of <0.2 and one considerably higher value. The possible reasons for this are currently being further investigated as are the reasons for occasional discrepancies in repeat determinations of CK-BB values.

Many of the cell lines have been continuously passaged in culture for 12–24 months (50–100 passages) following initial characterisation. In none of them has any gross change in culture morphology (e.g. to adeno or squamous cell carcinoma) been observed and the 'tightness of aggregation' (see Table IV) has been very stable. No repeat ultrastructural studies have been carried out but determinations of L-dopa decarboxylase activity carried out at later passages have been found to give values similar to those originally obtained.

### Discussion

Establishment in culture of cell lines from patients with small cell anaplastic carcinoma of the lung has been previously reported (Gazdar *et al.*, 1980; Pettengill *et al.*, 1980). Both of these groups used tissue culture medium containing 20% foetal calf serum. The lines obtained by the NCI group (Gazdar *et al.*, 1980) all grew as floating aggregates of cells whereas those of the Dartmouth group (Pettengill *et al.*, 1980) grew both attached to plastic or free floating or with a mixture of the two. In both reports, all small cell lines were found to have dense core vesicles (neurosecretory granules) when examined by electron microscopy. Furthermore, in the work of Gazdar *et al.* (1980) all lines were found to have relatively high levels of the enzyme DDC, considered to be a marker of APUD cells. More recently, the NCI group have described the use of a serum-free medium (HITES) (Carney *et al.*, 1981) for selective growth of SCLC cells. Some SCLC lines have been found to possess 'variant' morphology and to lack dense core granules and the enzyme DDC. In addition, the doubling time of such lines was reduced in comparison with 'classic' SCLC and the cloning efficiency in soft agar was higher (Carney *et al.*, 1984). In addition to these 'morphological variants' a further category of 'biochemical variants' has been found in which the cells show classic SCLC morphology but the biochemical changes associated with 'morphological variants'. Both variant types have high levels of CK-BB. The appearance of growing aggregates of cells has additionally been categorised into four types (Carney *et al.*, 1985; Gazdar *et al.*, 1985).

In the present study we have characterised 19 cell lines and believe that 14 of them are SCLC lines. There is very considerable heterogeneity among the lines. All four types of aggregation pattern are represented although some lines show a mixture of types. In general, levels of CK-BB were high in all the SCLC lines but an occasional relatively low value was seen. This observation is therefore in accordance with the data of Gazdar *et al.* (1981) in which high values of CK-BB were seen in SCLC lines. The content of DDC was very variable between lines. Of the three lines giving low values of DDC, two were examined by electron microscopy. One line (COR-L27) was found to show no evidence of neuroendocrine differentiation whilst another (COR-L80) showed only minimal differentiation. All three lines however possessed typical small cell cytological characteristics. They would therefore appear to fall into the category of 'biochemical' variants, by the NCI classification.

We have not attempted in this study to use population doubling time in culture or cloning efficiency in soft agar as determinants of SCLC culture type. It appears likely that the population doubling time is a secondary characteristic of the type of aggregates which different lines form, with lines showing very tight aggregates a higher proportion of non-cycling cells present. We are currently investigating this point. Furthermore, we believe that the low cloning efficiency claimed for SCLC by Gazdar *et al.* (1980) results from the use of non-optimal cloning conditions. We have found a plating efficiency of ~40% for line NCI-H69 compared with the value of 2.5% originally reported by Gazdar *et al.* (1980), and several of the SCLC lines described in the present paper have cloning efficiencies in excess of 20% in the assay of Courtenay & Mills (1978) (Twentyman & Walls, 1984; Walls & Twentyman, 1985).

It is interesting that line COR-L32 bearing numerous SCLC characteristics evidently arose from the marrow of a patient with poorly differentiated squamous cell carcinoma. The possibility of cross-contamination of cultures can rarely be definitively ruled out but COR-L32 differs in a number of ways from other SCLC lines isolated at around the same time. Current thought generally favours the concept that the various histological types of lung carcinoma form a differentiation continuum (Goodwin *et al.*, 1983) and it is not impossible therefore that a tumour developing as a squamous primary may show different characteristics following metastasis to the marrow and growth in culture.

A major problem in initiating tumour cell lines from biopsy material is that of fibroblast overgrowth. We have found that splitting initial samples between HITES and HITES+2.5% FCS provides an excellent approach to this problem. Of the lines reported here only COR-L23 was established in HITES alone but not in HITES +2.5% FCS (this line was not given the opportunity to establish in HITES+2.5% FCS). This is interesting in that HITES medium has been described as specific for SCLC (Carney *et al.*, 1981) whereas COR-L23 is a non-small cell line. All other lines established in HITES alone were also established in HITES+2.5% FCS. We do however have two more recent sets of lines (not reported in this paper) where the lines established in HITES alone appear on preliminary observation to be SCLC whilst the lines established in HITES+2.5% FCS appear to be B-lymphoblastoid. Such pairs of lines have a number of potential roles in experimental studies of antibodies directed against SCLC-associated antigens. We would suggest, however, that in cases where the number of cells available was very limited, initial culture in HITES +2.5% FCS with the option of switching to HITES alone for a period of time if fibroblasts become established is probably the best option.

Initially, all marrow samples were cultured and observed for a period of 6 months despite negative pathology reporting. This means a very large investment of time and effort when the production of lines from such samples is very rare. Unless one is particularly interested in validating the significance of negative marrow pathology it is probably not a worthwhile effort. Growth of lines from positive marrows, lymph node biopsies and pleural effusions is, however, successful in sufficient cases to justify the effort. It should of course be realised that these are all metastatic sites and the lines produced from such samples may not necessarily be characteristic of the primary tumour in the lung. Establishment of lines from bronchoscopy specimens is fraught with difficulty. The major difficulties are (a) the ethical justification for re-bronchoscoping a patient in order to obtain a sample for culture at some time after the original diagnostic biopsy; (b) the very small size of the specimen (which is frequently crushed) and (c) the likelihood of bacterial or fungal contamination. We have attempted to culture a number of bronchoscopy specimens subsequent to those listed in this paper and have only one still growing although not yet established as a line. The NCI group apparently have also had very limited success in this direction (Dr D. Carney – personal communication). The importance of establishing lines from primary SCLC is however so great that we believe that continued effort in this direction is essential.

We are currently engaged in a wide range of experimental studies using the lines described in this paper. These include derivation and characterisation of monoclonal antibodies to SCLC surface antigens (Reeve *et al.*, 1985), detailed examination of chromosome abnormalities, studies of radiation and cytotoxic drug response, tumour cell heterogeneity and mechanism of drug resistance.

We are indebted to Drs D. Carney and A. Gazdar of the Navy/NCI Medical Oncology Branch for advice and assistance on several occasions and to Dr Gazdar for giving his opinion on a large number of cytology preparations. Dr P. Stovin, Department of Pathology, Addenbrooke's Hospital, Cambridge, also kindly examined a number of slides. We are also grateful to Dr R. Thompson of the Department of Clinical Biochemistry, University of Cambridge, for carrying out the CK-BB analyses.

### References

ANNER, R.M. & DREWINKO, B. (1977). Frequency and significance of bone marrow involvement by metastatic solid tumours. *Cancer,* **39,** 1337.

BAYLIN, S.B., STEVENS, S.A. & SHAKIR, K.M.M. (1978). Association of diamine oxidase and ornithine decarboxylase with maturing cells in rapidly proliferating epithelium. *Biochem. Biophys. Acta.,* **541,** 415.

BAYLIN, S.B., ABELOFF, M.D., GOODWIN, G., CARNEY, D.N. & GAZDAR, A.F. (1980). Activities of L-DOPA decarboxylase and diamine oxidase (histaminase) in human lung cancers and decarboxylase as a marker for small (oat) cell cancer in cell culture. *Cancer Res.,* **40,** 1990.

BAYLIN, S.B., GAZDAR, A.F., MINNA, J.D., BERNAL, S.D. & SHAPER, J.H. (1982). A unique cell-surface protein phenotype distinguishes human small-cell from non-small-cell lung cancer. *Proc. Natl. Acad. Sci.* (USA), **79,** 4650.

BEAVEN, M.A., WILCOX, G. & TERPSTRA, G.K. (1978). A micro-procedure for the measurement of $^{14}CO_2$ release from [$^{14}C$] carboxyl-labelled amino acids. *Anal. Biochem.,* **84,** 638.

BECKER, K.L. & GAZDAR, A.F. (1983). The pulmonary endocrine cell and the tumors to which it gives rise. In *Comparative Respiratory Tract Carcinogenesis,* Vol. II, Reznik-Schiller (ed) p. 161. CRC Press: Boca Raton, Florida.

CARNEY, D.N., BUNN, Jr., P.A., GAZDAR, A.F., PAGAN, J.A. & MINNA, J.D. (1981). Selective growth in serum-free hormone-supplemented medium of tumor cells obtained by biopsy from patients with small cell carcinoma of the lung. *Proc. Natl. Acad. Sci.* (USA), **78,** 3185.

CARNEY, D.N., BRODER, L., EDELSTEIN, M., GAZDAR, A.F., HANSEN, M., HAVEMANN, K., MATTHEWS, M.J., SORENSON, G.D. & VIDELOV, L. (1983*a*), Experimental studies of the biology of human small cell lung cancer. *Cancer Treat. Rep.,* **67,** 27.

CARNEY, D.N., MITCHELL, J.B. & KINSELLA, T.J. (1983*b*). *In vitro* radiation and chemotherapy sensitivity of established cell lines of human small cell lung cancer and its large cell morphological variants. *Cancer Res.,* **43,** 2806.

CARNEY, D.N., NAU, M.M. & MINNA, J.D. (1984). Variability of cell lines from patients with small cell lung cancer. In *Human Tumour Cloning,* Salmon, S.E. *et al.* (ed) p. 67. Grune and Stratton, Orlando, Fla.

CARNEY, D.N., GAZDAR, A.F., BEPLER, G., GUCCION, J.G., MARANGOS, P.J., MOODY, T.W., ZWEIG, M.H. & MINNA, J.D. (1985). Establishment and identification of small cell lung cancer cell lines having classic and variant features. *Cancer Res.* (in press).

COURTENAY, V.D. & MILLS, J. (1978). An *in vitro* colony assay for human tumours grown in immune-suppressed mice and treated *in vivo* with cytotoxic agents. *Br. J. Cancer,* **37,** 261.

GAZDAR, A.F., CARNEY, D.N., RUSSELL, E.K., SIMS, H.L., BAYLIN, S.B., BUNN, Jr., P.A., GUCCION, J.G. & MINNA, J.D. (1980). Establishment of continuous, clonable cultures of small-cell carcinoma of the lung which have amine precursor uptake and decarboxylation cell properties. *Cancer Res.,* **40,** 3502.

GAZDAR, A.F., ZWEIG, M.H., CARNEY, D.N., VAN STEIRTEGHEN, A.C., BAYLIN, S.B. & MINNA, J.D. (1981). Levels of creatine kinase and its BB isoenzyme in lung cancer specimens and cultures. *Cancer Res.*, **41**, 2773.

GAZDAR, A.F., CARNEY, D.N., NAU, M.M. & MINNA, J.D. (1985). Characterisation of variant subclasses of cell lines derived from small cell lung cancer having distinctive biochemical, morphological and growth properties. *Cancer Res.* (in press).

GOODWIN, G., SHAPER, J.H., ABELOFF, M.D., MENDELSOHN, G. & BAYLIN, S.B. (1983). Analysis of cell surface proteins delineates a differentiation pathway linking endocrine and nonendocrine human lung cancers. *Proc. Natl. Acad. Sci.* (USA), **80**, 3807.

IHDE, D.C., SIMMS, E.B., MATTHEWS, M.J., COHEN, M.H., BUNN, P.A. & MINNA, J.D. (1979). Bone marrow metastases in small cell carcinoma of the lung: frequency, description and influence on chemotherapeutic toxicity and prognosis. *Blood*, **53**, 677.

JACKSON, A.P., SIDDLE, K. & THOMPSON, R.J. (1984). Two-site monoclonal antibody assays for human heart- and brain-type creatine kinase. *Clin. Chem.*, **30**, 1157.

KAMEYA, T., KODAMA, T. & SHIMOSATO, Y. (1982). Ultrastructure of small-cell carcinoma of the lung (oat and intermediate cell types) in relation to histogenesis and to carcinoid tumor. In *Morphogenesis of Lung Cancer*, Vol. II Shimosato, Y. *et al.* (ed) p. 15. CRC Press, Boca Raton, Florida.

LITTLE, C.D., NAU, M.M., CARNEY, D.N., GAZDAR, A.F. & MINNA, J.D. (1983). Amplification and expression of the c-myc oncogene in human lung cancer cell lines. *Nature*, **306**, 194.

LOWRY, O.H., ROSEBROUGH, N.J., FARR, A.L. & RANDALL, R.J. (1951). Protein measurement with the folin phenol reagent. *J. Biol. Chem.*, **193**, 265.

MARANGOS, P.J., GAZDAR, A.F. & CARNEY, D.N. (1982). Neuron specific enolase in human cell carcinoma cultures. *Cancer Lett.*, **15**, 67.

MOODY, T.W., PERT, C.B., GAZDAR, A.F., CARNEY, D.N. & MINNA, J.D. (1981). High levels of intracellular bombesin characterises human small-cell lung carcinoma. *Science*, **214**, 1246.

NILLSON, K. & KLEIN, G. (1982). Phenotypic and cytogenetic characteristics of human B-lymphoid cell lines and their relevance for the etiology of Burkitt's lymphoma. *Adv. Cancer Res.*, **37**, 319.

PETTENGILL, O.S., SORENSON, G.D., WURSTER-HILL, D.H., CURPHEY, T.J., NOLL, W.W., CATE, C.C. & MAURER, L.H. (1980). Isolation and growth characteristics of continuous cell lines from small-cell carcinoma of the lung. *Cancer*, **45**, 906.

REEVE, J.G., WULFRANK, D.A., STEWART, J., TWENTYMAN, P.R., BAILLIE-JOHNSON, H. & BLEEHEN, N.M. (1985). Monoclonal antibody-defined human lung tumour cell-surface antigens. *Int. J. Cancer*, **35**, 769.

SORENSON, G.D., PETTENGILL, O.S., BRINCK-JOHNSEN, T., CATE, C.C. & MAURER, L.H. (1981). Hormone production by cultures of small-cell carcinoma of the lung. *Cancer*, **47**, 1289.

SPURR, A.R. (1969). A low viscosity epoxy resin embedding medium for electron microscopy. *J. Ultrastruct. Res.*, **26**, 31.

TAYLOR, I.W. (1980). A rapid single step staining technique for DNA analysis by flow micro-fluorimetry. *J. Histochem. Cytochem.*, **28**, 1021.

TWENTYMAN, P.R. & WALLS, G.A. (1984). Factors influencing the clonogenicity of human lung cancer cells. In *Human Tumour Cloning*, Salmon, S.E. (ed) p. 279. Grune and Stratton, Orlando, Fla.

WALLS, G.A. & TWENTYMAN, P.R. (1985). Cloning of human lung cancer cells. *Br. J. Cancer*, **52**, 505.

WATSON, J.V. (1980). Enzyme kinetic studies in cell populations using fluorogenic substrates and flow cytometric techniques. *Cytometry*, **1**, 143.

WHANG-PENG, J., KAO-SHAN, C.S., LEE, E.C., BUNN, Jr., P.A.,. CARNEY, D.N., GAZDAR, A.F. & MINNA, J.D. (1982). A specific chromosome defect associated with human small cell lung cancer: deletion 3p (14–23). *Science*, **215**, 181.